# Integration of tissue metabolomics, transcriptomics and immunohistochemistry reveals *ERG-* and gleason score-specific metabolomic alterations in prostate cancer

**DOI:** 10.18632/oncotarget.6370

**Published:** 2015-11-23

**Authors:** Sebastian Meller, Hellmuth-A Meyer, Bianca Bethan, Dimo Dietrich, Sandra González Maldonado, Michael Lein, Matteo Montani, Regina Reszka, Philipp Schatz, Erik Peter, Carsten Stephan, Klaus Jung, Beate Kamlage, Glen Kristiansen

**Affiliations:** ^1^ Institute of Pathology, University Hospital of Bonn, Bonn, Germany; ^2^ Campus Wilhelminenhof, University of Applied Sciences, Berlin, Germany; ^3^ Metanomics GmbH, Berlin, Germany; ^4^ Berlin Institute for Urologic Research, Berlin, Germany; ^5^ Department of Urology, University Teaching Hospital, Offenbach, Germany; ^6^ Institute of Pathology, University of Bern, Bern, Switzerland; ^7^ Metanomics Health GmbH, Berlin, Germany; ^8^ Department of Urology, University Hospital Charité, Berlin, Germany

**Keywords:** prostate cancer, metabolites, ERG translocation, cholesterol, fatty acid

## Abstract

Integrated analysis of metabolomics, transcriptomics and immunohistochemistry can contribute to a deeper understanding of biological processes altered in cancer and possibly enable improved diagnostic or prognostic tests. In this study, a set of 254 metabolites was determined by gas-chromatography/liquid chromatography-mass spectrometry in matched malignant and non-malignant prostatectomy samples of 106 prostate cancer (PCa) patients. Transcription analysis of matched samples was performed on a set of 15 PCa patients using Affymetrix U133 Plus 2.0 arrays. Expression of several proteins was immunohistochemically determined in 41 matched patient samples and the association with clinico-pathological parameters was analyzed by an integrated data analysis. These results further outline the highly deregulated metabolism of fatty acids, sphingolipids and polyamines in PCa. For the first time, the impact of the *ERG* translocation on the metabolome was demonstrated, highlighting an altered fatty acid oxidation in *TMPRSS2*-*ERG* translocation positive PCa specimens. Furthermore, alterations in cholesterol metabolism were found preferentially in high grade tumors, enabling the cells to create energy storage. With this integrated analysis we could not only confirm several findings from previous metabolomic studies, but also contradict others and finally expand our concepts of deregulated biological pathways in PCa.

## INTRODUCTION

Prostate cancer continues to be the most frequent cancer in men with a predicted incidence of 220,800 new cases and 27,540 estimated deaths for 2015 in the US alone [1]. Intense research efforts aim to clarify the underlying molecular, physiological and biochemical processes of tumor initiation and progression of prostate cancer and “*-omics*” technologies such as genomics, transcriptomics, proteomics and metabolomics provide promising and holistic research tools [2].

Metabolomics is defined as a global quantitative or semi-quantitative analysis of all metabolites in a biological system such as an organ, tissue or body fluid. It has become a promising *-omics* approach applying the analytical platforms of nuclear magnetic resonance spectroscopy or mass spectrometry in combination with gas chromatography/liquid chromatography [3]. Metabolomics can be a valuable tool to comprehensively understand or monitor functional alterations of the cell, caused by alterations of preceding biological strata, i.e. by genomic, transcriptomic or proteomic changes. Moreover, an integrated view in combination with protein expression and gene expression data can further improve the understanding of regulatory mechanisms altered in the cancer cell.

Prostate cells have a distinct metabolic profile reflecting the production of citrate, prostate specific antigen (PSA) and polyamines that are major components of prostate fluid. When prostate cells undergo neoplastic transformation they lose the capacity to accumulate zinc, which leads to restored activity of the mitochondrial enzyme m-aconitase and thus citrate oxidation [4]. Consequently citrate levels decrease and ATP generation is increased. Furthermore, a number of key enzymes involved in fatty acid and cholesterol synthesis are upregulated in prostate cancer cells [5]. The alterations seen in the prostate cancer metabolome may be of diagnostic or therapeutic use (see recent review [6]). For example, the ratios (total choline+creatine+polyamines)/citrate (CCP/C) or (total choline+creatine)/citrate (CC/C), which are both increased in prostate cancer, are already used in magnetic resonance spectroscopic imaging (MRSI) and are thus implemented into clinical practice [7-9, 9].

Aims of this exploratory retrospective study, which complies to the recommendations of Early Detection Research Network [10], were: (a) to identify metabolites with different levels in malignant and non-malignant tissue, (b) to correlate these metabolites with conventional clinical-pathological variables (tumor stage and grade and *TMPRSS2*-*ERG* (*ERG*) translocation), and (c) to integrate these metabolites with a targeted protein expression analysis data set in order to identify regulatory or deregulatory mechanisms that were not obvious from a single –*omics* data set alone. A strength is the analysis of matched malignant and normal adjacent prostate cancer tissue from each subject that enabled a statistical correction for inter-individual variability of the metabolite data.

## RESULTS

### Patient characteristics

For metabolite analysis, 106 malignant and matched normal adjacent tissue (NAT) samples from prostate cancer patients with complete clinical characteristics were collected. Additionally, protein expression was analyzed in 41 of these patient samples and transcription profiling was determined in 15 other patient samples (Table 1).

**Table 1:** Clinical characteristics of the study group

Characteristics	Total (MxP^®^ Broad Profiling)	Protein expression data	Transcription data
Cohort size	106	41	15
Age, years			
Mean (SD^1^)	62 (6.5)	63 (5.9)	60
Range	46-73	46-73	47-69
Body Mass Index, kg/m^2^			
Mean (SD^1^)	26 (3.5)	27(3.6)	n.a.^2^
Range	19-37	21-37	n.a.^2^
No. pathological stage (%)			
pT2	65 (61)	26 (63)	10 (67)
pT3	41 (39)	15 (27)	5 (33)
Margin R (%)			
0	62 (58)	27 (66)	10 (66.7)
1	43 (41)	14 (34)	4 (26.7)
n.a.^2^	1 (1)	0 (0)	1 (6.7)
No. ERG translocation (%)			
positive	27 (25)	18 (44)	
negative	23 (22)	13 (32)	
n.a.^2^	56 (53)	10 (24)	15 (100)
Gleason Score (%)			
<7	33 (31)	17 (42)	3 (20)
=7	44 (42)	14 (34)	9 (60)
>7	29 (27)	10 (24)	3 (20)
Biochemical recurrence			
Follow-up months, mean	55	n.a.	n.a.
Recurrence (%)	21 (20)	n.a.	n.a.
No recurrence (%)	74 (70)	n.a.	n.a.

1 SD= Standard deviation;

2 n.a. not analyzed

### Metabolite levels correlate with Gleason score and ERG translocation

A total of 254 metabolites with 172 known and 82 unknown spectral features were found. 134 of the known metabolites and 39 of the unknowns showed differential levels in malignant versus non-malignant samples (Table 2). Of these 134 known metabolites, 92 were at least 1.2 fold increased and eight metabolites were at least 0.83 fold decreased (Supplemental Table 1). Among them were several metabolites already considered to be involved in prostate cancer such as sarcosine, polyamines or cholines [11] (Supplemental Table 1). No metabolites showed a significant change (p<0.05 and false-positive-discovery rate <0.2) associated with body mass index (BMI). An analysis of the influence of pT category (pT2 versus pT3 tumors) on the metabolome did not reveal significant alterations (Table 2). Unsupervised cluster and principal component analysis of 76 same-subject samples (≤10% missing values) provided evidence of a metabolic distinction between malignant and normal adjacent tissue (Supplemental Figure 1).

**Table 2:** Number and percentage of significant metabolite changes out of 254 metabolites of 106 matched malignant and adjacent normal prostate tissue samples Statistical analysis was done via mixed ANOVA models; the significance level was set to *p*<0.05 and FDR<0.2.

Analysis	Metabolite ontology	Number of significantly changed metabolites
Malignant versus adjacent normal	**Increased**	**156**
	Amino acids	20
	Amino acids related	9
	Carbohydrates and related	3
	Complex lipids, fatty acids and related	54
	Energy metabolism and related	7
	Miscellaneous	7
	Nucleobases and related	9
	Vitamins, cofactors and related	11
	Unknown*	36
	**Decreased**	**17**
	Amino acids	1
	Carbohydrates and related	6
	Complex lipids, fatty acids and related	3
	Energy metabolism and related	1
	Miscellaneous	2
	Nucleobases and related	1
	Unknown*	3
Gleason score	**Increased**	**11**
	Amino acids related	1
	Complex lipids, fatty acids and related	5
	Nucleobases and related	1
	Vitamins, cofactors and related	1
	Unknown*	3
	**Decreased**	**4**
	Carbohydrates and related	2
	Complex lipids, fatty acids and related	1
	Energy metabolism and related	1
ERG Translocation	**Increased**	**53**
	Amino acids Amino acids related Carbohydrates and related	14 2 1
	Complex lipids, fatty acids and related	13
	Nucleobases and related Vitamins, cofactors and related	6 4
	Unknown*	13
	**Decreased**	**17**
	Amino acids related Carbohydrates and related Energy metabolism and related Miscellaneous Nucleobases and related Unknown*	2 8 2 2 1 2
pT3 versus pT2	-	0
BMI	-	0

* Metabolites with final chemical structure pending

With regard to the Gleason score, eight known and four unknown metabolites were up-regulated, whereas four metabolites were down-regulated with increasing Gleason score (Table 2, Table 3A) applying a mixed linear model (ANOVA) with Gleason score as a numerical factor. For example, increased Gleason score was positively correlated with pantothenic acid, a constituent of coenzyme A and the acyl carrier protein. Maltose, fructose-6-phosphate, gluconic acid, and cholesterol were negatively correlated with the Gleason score. Gluconic acid has been reported before by us in an analysis of nine selected metabolites [12].

**Table 3A:** Overview of ANOVA results of all significantly changed metabolites with Gleason score, unknowns not included ANOVA analysis is described in the method section. Ratio gives the fold change of the metabolite when Gleason score increases by one SD

Metabolite name	Ratio	*p*-value	Ontology name
14-Methylhexadecanoic acid	1.18	1.25E-03	Complex lipids, fatty acids and related
Myristic acid (C14:0)	1.15	8.02E-03	Complex lipids, fatty acids and related
Uracil	1.13	6.50E-04	Nucleobases and related
Pentadecanol	1.12	9.29E-03	Fatty alcohols
Heptadecanoic acid (C17:0)	1.11	5.90E-03	Fatty acids, saturated
Pantothenic acid	1.11	9.15E-03	Acyl-carriers and related
Isopentenyl pyrophosphate (IPP)	1.10	1.07E-02	Mevalonate pathway
Homogentisic acid	1.08	1.22E-02	Amino acid metabolites
Cholesterol, total	0.94	9.94E-03	Cholesterol and related
Fructose-6-phosphate	0.85	6.13E-03	Glycolysis/Gluconeogenesis
Maltose	0.80	5.86E-03	Disaccharides
Gluconic acid (additional: Gluconolactone)	0.80	8.43E-03	Sugar acids

**Figure 1:**
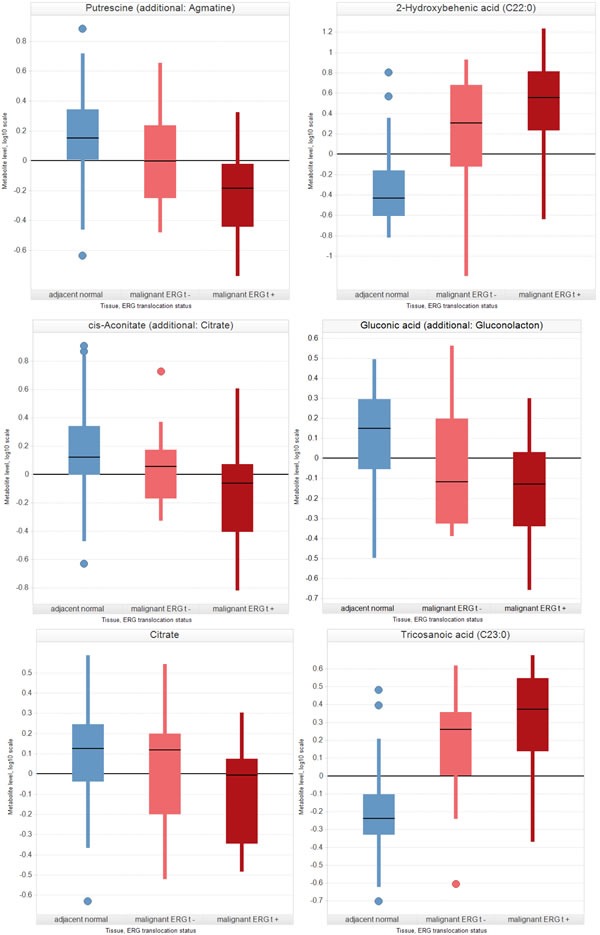

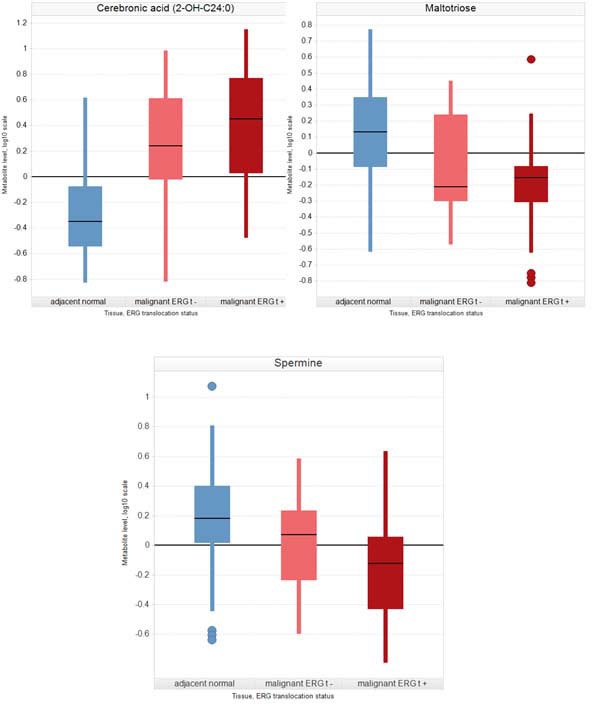
Metabolic fingerprint of the ERG translocation in prostate cancer tissue Box plot represents a plot of 5 parameters, i.e. median, lower quartile (Q1), upper quartile (Q3), upper adjacent value, lower adjacent value and, if present, additionally display outliers. Adjacent values: Let IQR be the interquartile range (Q3 – Q1). The upper adjacent value (upper whisker) is the largest observation that is less than or equal to the upper inner fence (UIF) which is the third quartile plus 1.5*IQR. The lower adjacent value (lower whisker) is the smallest observation that is greater than or equal to the lower inner fence (LIF), which is the first quartile minus 1.5*IQR. Outliers are values that fall outside the whiskers. Expression levels (subject-corrected residuals) of selected metabolites that are significantly regulated by ERG translocation with *p*<0.05 and FDR<0.2. *P*-values are given in Table 3B.

In samples of prostate cancer with an *ERG* translocation, 40 known and 15 unknown metabolites were found to be increased and 15 known and two unknown metabolites were found to be decreased in comparison to *ERG*-negative carcinomas (Table 2, Table 3B). *ERG* translocation was negatively correlated with maltotriose and gluconic acid, that have already been negatively associated with prostate cancer recurrence [12]. The three fatty acids with the highest levels were cerebronic acid (2-OH-C24:0), 2-hydroxybehenic acid (C22:0), and tricosanoic acid (C23:0). Interestingly, citrate and cis-aconitate and also the polyamines spermine and putrescine were significantly decreased in *ERG*-positive samples (Figure 1, Table 3B).

**Table 3B:** Overview of ANOVA results of all significantly changed metabolites with ERG translocation, unknowns not included ANOVA analysis is described in the method section. Ratio gives the fold change of ERG translocation positive versus ERG translocation negative samples

Metabolite name	Ratio	*p*-value	Ontology name
2-Hydroxybehenic acid (C22:0)	3.99	5.10E-05	Complex lipids, fatty acids and related
Cerebronic acid (2-OH-C24:0)	3.24	1.75E-04	Complex lipids, fatty acids and related
Cystine	2.82	1.12E-04	Amino acids
Tricosanoic acid (C23:0)	2.33	5.50E-05	Complex lipids, fatty acids and related
Xanthine	1.82	1.19E-03	Nucleobases and related
Eicosadienoic acid (C20:2) No 02	1.60	2.93E-03	Complex lipids, fatty acids and related
Docosapentaenoic acid (C22:cis[7, 10, 13, 16, 19]5)	1.57	2.80E-05	Complex lipids, fatty acids and related
7-Methylguanine	1.55	2.53E-04	Nucleobases and related
Isopentenyl pyrophosphate (IPP)	1.55	6.40E-04	Complex lipids, fatty acids and related
erythro-Dihydrosphingosine (d16:0)	1.51	1.20E-02	Complex lipids, fatty acids and related
Cysteine (additional: Cystine)	1.47	9.90E-05	Amino acids
Glycerophosphoethanolamine, polar fraction	1.46	4.70E-02	Complex lipids, fatty acids and related
Heptadecanoic acid (C17:0)	1.40	6.58E-03	Complex lipids, fatty acids and related
gamma-Tocopherol	1.39	1.40E-02	Vitamins, cofactors and related
Uracil	1.38	3.00E-05	Nucleobases and related
Methionine	1.37	4.44E-04	Amino acids
Histidine	1.36	1.94E-02	Amino acids
Uridine	1.36	3.11E-02	Nucleobases and related
Pantothenic acid	1.34	6.73E-03	Vitamins, cofactors and related
Biotin	1.32	5.45E-03	Vitamins, cofactors and related
Hypoxanthine (additional: Inosine)	1.31	3.77E-02	Nucleobases and related
Threonic acid	1.31	6.35E-03	Vitamins, cofactors and related
Linoleic acid (C18:cis[9, 12]2)	1.31	3.47E-03	Complex lipids, fatty acids and related
Glycine	1.27	4.02E-03	Amino acids
Aspartate	1.27	1.48E-02	Amino acids
Sphingomyelin (d18:1,C23:0)	1.26	8.10E-05	Complex lipids, fatty acids and related
Proline	1.25	2.03E-02	Amino acids
Ribose	1.24	3.49E-02	Carbohydrates and related
trans-4-Hydroxyproline	1.23	1.70E-02	Amino acids related
Arginine	1.22	2.08E-02	Amino acids
Tyrosine	1.21	3.60E-02	Amino acids
Isoleucine	1.20	1.11E-02	Amino acids
Leucine	1.19	3.14E-02	Amino acids
Phenylalanine	1.19	3.98E-02	Amino acids
Cytosine (additional: 2′-Deoxycytidine)	1.16	5.83E-03	Nucleobases and related
Lysophosphatidylcholine (C18:2)	1.16	4.15E-02	Complex lipids, fatty acids and related
Tryptophan	1.16	4.28E-02	Amino acids
Glutamate	1.16	4.10E-02	Amino acids
5-Oxoproline (additional: Folic acid, Glutamate, Glutamine)	1.14	3.09E-02	Amino acids related
Ceramide (d18:1,C24:1) (additional: Ceramide (d18:2,C24:0))	1.09	2.59E-02	Complex lipids, fatty acids and related
myo-Inositol	0.86	4.37E-02	Carbohydrates and related
Creatinine	0.85	3.86E-02	Amino acids related
scyllo-Inositol	0.85	1.21E-02	Carbohydrates and related
Creatine	0.83	1.08E-03	Amino acids related
Adenosine monophosphate (AMP)	0.75	3.76E-02	Nucleobases and related
Mannose	0.66	1.89E-02	Carbohydrates and related
Maltose	0.65	2.53E-02	Carbohydrates and related
Citrate	0.65	3.64E-02	Energy metabolism and related
Glucuronic acid	0.62	1.92E-02	Carbohydrates and related
Glucose	0.61	2.81E-02	Carbohydrates and related
cis-Aconitate (additional: Citrate)	0.58	3.30E-02	Energy metabolism and related
Maltotriose	0.56	2.98E-02	Carbohydrates and related
Gluconic acid (additional: Gluconolacton)	0.53	1.84E-03	Carbohydrates and related
Spermine	0.48	9.97E-03	Miscellaneous
Putrescine (additional: Agmatine)	0.36	3.30E-05	Miscellaneous

### Cox proportional hazards analysis

In order to evaluate the prognostic potential of metabolites from prostate cancer we performed Cox proportional hazards analysis for biochemical recurrence after prostatectomy. Among the top ten separating metabolites were nine amino acids or amino acid related metabolites and one fatty acid (Table 3C). Given the numbers of 36 amino acid or amino acid related metabolites and 68 lipids in the data set, this is apparently an enrichment of amino acid or amino acid related metabolites. The pT value showed the highest hazard ratio with an only marginally higher p-value than the amino acids tryptophan and tyrosine.

**Table 3C:** Top ten metabolites and pathological state (pT) in Cox Hazard Ratio analysis of recurrance of prostate cancer ordered by p-value The statistical method is described in the method section. CI, confidence interval; FDR, false-discovery rate

Variable	Hazard Ratio (± 95% CI)	*p*-value	FDR
Tryptophan	2.17 (1.54, 3.05)	0.00001	0.0022
Tyrosine	1.88 (1.36, 2.59)	0.0001	0.0133
Pathological state	8.02 (2.69, 23.89)	0.0002	0.0133
Ornithine (additional: Arginine, Citrulline)	1.66 (1.27, 2.18)	0.0002	0.0133
Lysine	1.84 (1.32, 2.56)	0.0003	0.0157
Isoleucine	1.93 (1.34, 2.76)	0.0004	0.0157
Aspartate	1.73 (1.27, 2.35)	0.0005	0.0181
Threonine	1.67 (1.23, 2.26)	0.0010	0.0283
Valine	1.84 (1.28, 2.64)	0.0010	0.0283
Sarcosine	1.44 (1.15, 1.8)	0.0016	0.0418
Stearic acid (C18:0)	1.91 (1.27, 2.88)	0.0019	0.0451

### Integrated analysis of transcription, immunohistochemistry, and metabolomic data demonstrate an altered energy and lipid metabolism in malignant tissue

**Figure 2:**
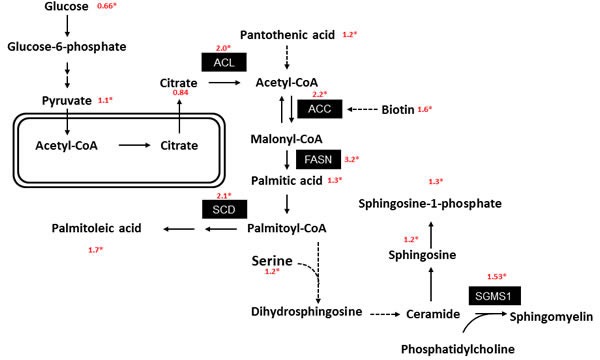
Prostate cancer metabolism is associated with increased fatty acid synthesis Pathway of energy and lipid metabolism: The corresponding mRNA as well as metabolite expression values (ratio: tumor/adjacent normal) were indicated. * indicates a significant level of p<0.05 and FDR<0.2 (for metabolites) and a corrected (Benjamini & Hochberg method) p<0.05 (for proteins and transcripts), respectively. ACL: ATP citrate synthase. ACC: Acetyl-CoA carboxylse. FASN: Fatty acid synthase complex. SCD: Acyl-CoA desaturase. SGMS1: Phosphatidylcholine:ceramide cholinephosphotransferase 1.

Significantly differentially regulated metabolites/genes discriminating between malignant and non-malignant tissues were used for network analysis. The role of fatty acid metabolism in prostate cancer was investigated in more detail. Several intermediates, products and involved enzymes were found to be altered in malignant tissue samples compared to benign ones (Table 2 and Supplemental Table 1). Malignant tissue samples showed an increased expression of acetyl-CoA carboxylase (ACC), ATP citrate lyase (ACL) and fatty acid synthase (FASN) which represent important enzymes in fatty acid biosynthesis or, in the case of ACL, provide a link to the metabolism of carbohydrates (Figure 2 and Supplemental Table 2). The amounts of the matching input and output metabolites such as citrate, pantothenic acid or biotin were significantly altered in the prostate cancer samples compared to the non-malignant samples. The upregulation of palmitic acid together with the higher expression of acyl-CoA desaturase (SCD) both lead to a higher level of the monounsaturated fatty acid palmitoleic acid. Palmitic acid is one educt of sphingolipid *de novo* biosynthesis (Figure 2).

**Figure 3:**
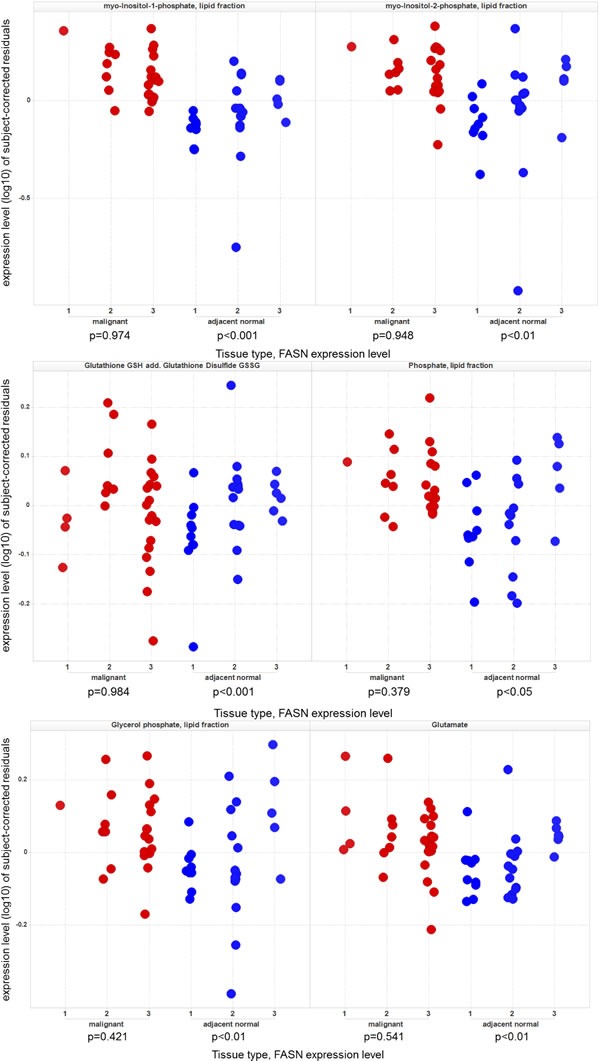
Expression levels (subject-corrected residuals) of selected metabolites in correlation to FASN protein expression in both prostate cancer (malignant) tissue and the corresponding adjacent normal tissue Only in normal adjacent tissue FASN protein expression correlates with the level of metabolites. P-values are from tissue-type-specific ANOVA with FASN protein expression, age, BMI and storage time as numerical factors.

Correlation analysis (Spearman) between protein expression and the metabolite profiles revealed a significant correlation only for FASN if the false-positive discovery rate is taken into consideration, with several metabolites in NAT (Supplemental Figure 2). Several fatty acids such as 2-hydroxybehenic acid, cerebronic acid, or glycerol phosphate in the lipid fraction (which is therefore a lipid fragment and can be considered a sum parameter of all glycerol-phospholipids) showed significantly higher concentrations in malignant tissues (Supplemental Table 1). This was consistent with the increased expression of FASN, both on mRNA and on protein levels in prostate cancer (Figure 2 and Supplemental Table 2). However, in contrast to benign tissues a significant correlation between the expression level of FASN and the concentration of fatty acids was not found in tumor tissues (Figure 3) thereby indicating a deregulation of this metabolite-enzyme correlation in cancer metabolism.

### Polyamine, glutathione, and myo-inositol metabolism is changed in prostate cancer

Though most metabolites were upregulated in prostate cancer compared to normal tissues, we have also analyzed the few decreased ones. Several metabolites involved in polyamine metabolism were changed in prostate cancer. While putrescine and spermine were decreased, spermidine was increased (Supplemental Table 1). Furthermore, the important antioxidant glutathione (GSH) was increased in cancer samples. The oxidized state of GSH glutathione disulfide (GSSG) however, showed lower levels in malignant tissues (Supplemental Table 1). A decrease of myo-inositol from the polar fraction of the tissue and an increase of myo-inositol-1-phosphate from the lipid fraction (Supplemental Table 1) was also observed. Inositol-1-phosphate (lipid fraction) is an integrative measure of all inositol-1-phosphate containing lipids and is generated during the derivatization of the lipid extract prior to GC-MS analysis.

## DISCUSSION

The field of cancer metabolism has rapidly progressed in recent years. In contrast to earlier assumptions, the theory has emerged that altered metabolite concentrations may also causally promote tumor progression and are thus not merely a downstream effect of the characteristic neoplastic growth interfering with metabolism. However, many open questions remain: Which metabolic pathways are deregulated in which type of cancer and by which specific oncogenes? Which nutrients are essential? Finally, does diet influence cancer development and/or progression (reviewed in [13])? Earlier studies tackling the issue of prostate cancer metabolism lacked the power to answer these questions and often included either too few patient samples or used cell lines. In the present study, we performed global metabolite profiling of 106 matched malignant and non-malignant samples from the same patient. Furthermore, we conducted a transcription profiling study from an additional set of 15 prostate cancer patients. This study therefore comprises the largest cohort so far analyzed in addressing this issue. With this integrated analysis of metabolic, transcriptional and immunohistochemical data, we were not only able to confirm specific findings from previous metabolomic studies, but also to contradict others. Finally, and perhaps more importantly, we were able to extend our concept of the role of deregulated biological pathways in prostate cancer.

### Fatty acid and sphingolipid pathway

FASN expression and lipid metabolism is deregulated in malignant tissue. The strong correlation of FASN with the concentration of fatty acids was observed exclusively in benign prostate tissue. This could be due to the specific metabolism of the normal prostate. In normal ductal prostate cells high zinc levels block the Krebs cycle [4, 14] thereby compromising the oxidation of fatty acids. As a result, the content of fatty acids can be directly related to the level of FASN in normal tissue. This specific metabolic characteristic of ductal prostate cells is altered in transformed prostate cells [4, 14] leading to activation of the Krebs cycle. Since increased fatty acid metabolism characterizes prostate cancer [15], the correlation between FASN expression and fatty acid content vanishes. This is in accordance with a previous study by Svinnen *et al.* who also described this absence of a correlation between FASN and fatty acid content in prostate cancer samples [16]. This may further explain why [F-18]-fluorodeoxyglucose-positron emission tomography (FDG-PET), which detects cancer based on the Warburg effect, is limited to the detection of advanced prostate cancers [17].

As a result of the aberrantly activated Krebs cycle in prostate cancer cells, decreased citrate levels are to be expected. In the present study, citrate was indeed decreased in the malignant compared to the non-malignant tissue (ratio 0.82). Although this change was statistically non-significant, the cut-off was missed only narrowly (p=0.056). Despite citrate being a robust metabolite from an analytical point of view, an earlier study also revealed that a citrate decrease in prostate cancer tissue could not always be reproducibly detected in independent cohorts [18]. This might be due to the relatively small difference of citrate levels in cancer cells compared to normal cells on the one hand and/or high variance among patients on the other, both of which would impede the detection of statistically significant differences.

Our study contradicts the findings of Moore *et al*. 2005 [19], who reported SCD loss in prostate cancer, whereas our analyses showed an increase in the SCD expression at both the mRNA and protein level. This is further supported by the metabolomic data, which demonstrated increased levels of oleic acid, a product of SCD. Our results are in agreement with those from other authors who also reported an overexpression of SCD1 in prostate cancer cells [20].

### The metabolomic fingerprint of Gleason score

Higher Gleason scores are positively correlated with pantothenic acid, a constituent of coenzyme A and the acyl carrier protein, which are key factors of primary metabolism including the citrate cycle and fatty acid biosynthesis. The negative correlation of maltose and fructose-6-phosphate with the Gleason score could indicate increased catabolism of tumor cells. This might reflect the raised importance of glycolysis in hypoxic malignant prostate tissue as found predominantly in high grade cancers. This is in line with the significant positive correlations between Gleason score and the hypoxia markers glucose transporter-1 and lysyl oxidase as shown earlier [21].

### Cholesterol metabolism is altered in prostate cancers with high Gleason score

The levels of isopentenyl pyrophosphate (IPP), an intermediate of the HMG-CoA reductase pathway (mevalonate pathway), were positively correlated with Gleason score whereas cholesterol levels were inversely correlated. However, cholesterol levels in the malignant tissue were significantly higher than in the non-malignant tissue. This observation could point to the modification of cholesterol metabolism resulting from the increased aggressiveness of prostate cancer cells. SREBP (sterol regulatory element-binding protein) is a direct target of phosphatidylinositol-3 kinases/Akt (PI3K/Akt) and of mitogen-activated protein kinase (MAPK) pathways. SREBP senses low cholesterol levels and stimulates the expression of key enzymes or receptors (LDL-receptor) of lipid synthesis and uptake by the HMG-CoA-reductase pathway [22, 23]. Recently, Yue *et al*. [24] reported an accumulation of cholesteryl esters in the most aggressive cancer cells but not in normal prostate cells. The increased cholesteryl ester levels arose from the significantly enhanced uptake of exogenous lipoproteins, themselves induced by *PTEN* (phosphatase and tensin homolog) loss and PI3K/Akt activation. Normally when cholesterol is abundant in the cell, SREBPs are retained in the endoplasmic reticulum. When cholesterol levels decrease however, SREBPs are cleaved and act as transcription factors. ACAT (acyl coenzyme A cholesterol acyltransferase) converts a surplus of free cholesterol to cholesterol esters that accumulate in the cytoplasm as cholesteryl esters. It has been proposed that ACAT guards against excessive synthesis of cholesterol in the endoplasmic reticulum [25]. The deregulation of this mechanism in more aggressive tumors, as suggested by the findings of Yue *et al.* [24] and by those in the present study, could therefore lead to the artificial reduction of cholesterol levels in the endoplasmic reticulum and in doing so prevent inhibition of the cleavage/activation of SREBPs. In this way the cells would be able to maintain an energy store as cholesteryl esters while not interfering with SREBP-mediated lipid synthesis and uptake. This is in accordance with findings from previous studies in that myristic acid (increased with higher Gleason score) was found to increase cholesterol levels [26] and high serum levels were associated with an increased risk of prostate cancer [27].

### ERG translocation shows a fingerprint in metabolomics data

Although *ERG* translocation is considered characteristic of prostate cancers, its influence on the metabolome has as yet not been investigated. The present study showed that *ERG* translocation is negatively correlated with both maltotriose and gluconic acid. Decreased levels of these metabolites have already been reported to be associated with earlier prostate cancer (biochemical) recurrence [12]. Furthermore, we found a positive correlation between *ERG* translocation and tryptophan, tyrosine, isoleucine, and aspartate. Patients with elevated levels of these metabolites had a higher risk of biochemical recurrence. This finding is supported by the positive association of *ERG* translocation with an increased risk of progression during active surveillance [28] and in several watchful waiting cohorts [29, 30]. Furthermore, serum levels of sulfur-containing amino acids (increased in *ERG*-positive tumors), especially cysteine, were also shown to be associated with prostate cancer recurrence [31]. On the other hand, the significantly decreased citrate levels found in *ERG*-positive tumors suggest a role of *ERG* translocation in the initiation of prostate cancer, as both factors have been previously described as early events in tumorigenesis [4, 32, 33]. ERG overexpression may also lead to altered intracellular zinc concentration and/or distribution. In this way the switch from glycolytic to fatty acid metabolism (functional citrate metabolism) could be initiated and/or promoted by ERG. The significantly decreased levels of several sugars (glucose, mannose, maltose, maltotriose) and cis-aconitate together with a simultaneous increase of several fatty acids in *ERG*-positive tumors support this hypothesis. This is further underpinned by the observed increased levels of the zinc-binding amino acids cysteine, histidine, aspartate and glutamate. The increased levels of various nucleobases and amino acids could reflect an increased proliferation in *ERG*-positive tumors which may also be an effect of low zinc levels. The three fatty acids with the highest increase in *ERG*-positive tumors compared to *ERG*-negative tumors were cerebronic acid, 2-hydroxybehenic acid and tricosanoic acid (C23:0). These metabolites further showed the highest discriminative power between normal and cancerous prostate tissue [12]. As already reported by Jung *et al*. [12], cerebronic acid and 2-hydroxybehenic acid cannot be metabolized by β-oxidation but only by α-oxidation localized in the peroxisome. Cerebronic acid is decarboxylated by α-oxidation to tricosanoic acid and CO_2_ [34]. Peroxisomal fatty acid oxidation was reported to be upregulated in prostate cancer [35] and *ERG* translocation might promote this shift. Interestingly, we found a significant decrease of creatine in malignant samples, predominantly in *ERG*-positive tumors. Phosphocreatine, the phosphorylated counterpart of creatine, supports cells with energy in times of high energy demand [36]. It is synthesized from glycine, arginine and methionine which are all increased in the *ERG*-positive tumors, thus implying such a demand.

In summary, *ERG* translocation seems to affect energy metabolism of prostate cells with particular attention to fatty acid metabolism. This is in very good accordance with the results of Pettersson *et al*. [37] who showed that obesity is associated with poorer prognosis primarily in patients with *ERG*-positive tumors. Another important finding of our study was the negative correlation of spermine and putrescine with *ERG* rearrangement. Although polyamine down regulation in prostate cancer has already been described in several studies [38-40], until now it has never been contextualized in relation to *ERG* translocation.

Interestingly, amino acids dominated among the metabolites significantly associated with biochemical recurrance. This finding supports the idea of amino acid based PET for prostate cancer recurrence, as described for leucine and tryptophan by Hong *et al*. [41] and also lends a translational aspect to promote future directions of clinical applications. However, the superiority of the amino acid analysis over the pT score has to be validated in further studies.

Several limitations of the study need to be mentioned. To avoid a selection bias, the samples were used according to the availability of cryopreserved tissue in consecutive order. Multiple testing problems were addressed by calculating the false discovering rate. It has been shown that prostate cancer exhibits a “field effect” that influences the metabolome of the normal adjacent tissue [42, 43]. Therefore, matched normal tissue was sampled with maximum possible spatial discrimination from the direct vicinity of the carcinoma, ideally from a separate block. To be able to correct for inter-individual variability during statistical analysis of the metabolite data, it was also necessary to work with paired (same patient) samples (NAT and cancer), which is not possible when sampling prostate tissue from healthy patients. In addition, the availability of healthy prostate tissue is very limited. This field effect could also partly explain the often modest fold changes of the differentially-expressed metabolites and the borderline significance of citrate. The robustness of the results from this medium-sized unicentric cohort study requires external validation. With respect to the findings from the survival analyses, a limitation of our study is that we used prostatectomy specimens. The potential of medical application would be greatly increased by the transferability of the results to prostate biopsies and, although more challenging, to formalin-fixed paraffin embedded prostate biopsy specimens.

In summary, this integrated analysis further outlines the highly deregulated fatty acid and sphingolipid metabolism in prostate cancer. Furthermore, we could show that individual metabolites correlate with Gleason scores and for the first time the impact of the *ERG* translocation on the metabolome could be demonstrated. These data point to an altered cholesterol metabolism in more aggressive cancer types and an altered fatty acid oxidation in *ERG*-positive tumors. These findings imply that metabolomics may be able to more clearly characterize altered cellular networks and activity associated with disease states.

## MATERIALS AND METHODS

### Study design

Tumor tissue and matched normal adjacent tissue were taken from prostate specimens after radical prostatectomy between 2001 and 2007. For subsequent metabolite and expression analysis a full frontal tissues slice of 2-4 mm thickness was immediately cryopreserved in liquid nitrogen. Methodical details about the obtainment of pure tumor tissue (>90%) and matched adjacent normal tissue were described previously [44]. For each patient, clinicopathological information on age, body mass index, tumor classification according to the UICC 2002 TNM System, and tumor Gleason grade based on the whole specimen were compiled (Table 1). The study protocol was approved by the local ethical board.

### MxP^®^ Broad Profiling analysis

Two types of mass spectrometry analyses were applied to all samples. GC-MS (gas chromatography-mass spectrometry; Agilent 6890 GC coupled to an Agilent 5973 MS-System, Agilent, Waldbronn, Germany) and LC-MS/MS (liquid chromatography-MS/MS; Agilent 1100 HPLC-System (Agilent, Waldbronn, Germany) coupled to an Applied Biosystems API4000 MS/MS-System (Applied Biosystems, Darmstadt, Germany)) were used for MxP® Broad Profiling [45, 46]. The sample preparation process was optimized for this specific tissue type previously to enable this study. The fresh-frozen tissue material was freeze-dried and extracted with polar (water) and non-polar (ethanol/dichloromethane/acetonitrile) solvents. The extract was fractioned into an aqueous, polar phase (polar fraction) and an organic, lipophilic phase (lipid fraction). For GC-MS analyses, the non-polar fraction was treated with methanol under acidic conditions to yield the fatty acid methyl esters derived from both free fatty acids and hydrolyzed complex lipids. The polar and non-polar fractions were further derivatized with O-methyl-hydroxylamine hydrochloride (20 mg/ml in pyridine, 50 μl) to convert oxo-groups to O-methyloximes and with a silylating agent (N-Methyl-N-(trimethylsilyl) trifluoroacetamide, 50 μl) before GC-MS analysis. For LC-MS/MS analyses, both fractions were reconstituted in appropriate solvent mixtures. HPLC was performed by gradient elution using methanol/water/formic acid on reversed phase separation columns. A special mass spectrometric detection technology was applied, which allowed for targeted and high sensitivity multiple reaction monitoring (MRM) profiling in parallel with a full screen analysis. For the polar fraction, the instrument was operated in negative ionization mode, for the lipid fraction in positive ionization mode. Mass spectrometry detection was performed with repetitive cycles of MRM transitions for important pre-selected metabolites followed by a full scan from m/z of 100 to 1000.

### Data normalization, data set alignment, metabolite levels and nomenclature

Metabolite profiling based on a semi-quantitative analytical platform results in relative metabolite levels referenced to a defined control group (“ratio”). To support this concept, aliquots of pooled samples (= “pool”) generated from extra samples provided for this purpose were run in parallel throughout the whole process. For all semi-quantitatively analyzed metabolites, the data were normalized against the median in the pool reference samples within each analytical sequence to provide pool-normalized ratios (performed for each sample per metabolite). This process step compensated for inter- and intra-instrumental variation.

The limit of detection and the dynamic range of the semi-quantitative measurements were determined by dilution and spiking experiments during method development. In total, 254 metabolites were analyzed semi-quantitatively, 82 thereof being “unknown”, i.e. metabolites with known retention time and mass spectrum but chemical structure pending. The raw peak data were normalized to the sample weight and to the median of pool samples per analytical sequence to account for process variability (so called “ratios versus pool”).

A rigorous quality control was performed on peak, analyte and sample level. Within each analytical sequence, the signals of the internal standards were plotted onto control charts. Samples that displayed >30% standard deviation of one of the internal standards, were invalidated. Outlier peaks on group level (carcinoma tissue, control tissue) were identified by boxplot analyses, manually checked for correct annotation and integration and, if necessary, manually corrected. Peaks with very low metabolite abundance, e.g. that did not allow reliable peak integration or that did not meet requirements of retention time index, were not analyzed but converted to missing values.

Details on metabolite nomenclature are available [47] but in short, the term “additional” (add.) was applied to indicate that quantification can be disturbed by metabolites exhibiting identical analytical characteristics with respect to the quantitation method. Further, components of the lipid backbone (i.e. glycerol) were quantified in the non-polar phase (carrying the term “lipid fraction” following the metabolite name). For example, “glycerol, lipid fraction” represents glycerol liberated during the derivatization process from complex lipids – in contrast, “glycerol, polar fraction” represents glycerol present originally in the polar phase of the biological sample.

### Analysis of protein expression levels and ERG translocation

Protein expression levels were measured by immunohistochemistry on formalin-fixed and paraffin-embedded tissue samples. Immunohistochemical staining of the tissue sections were performed on the Leica BondMax (Bond; Labvision, Fremont, CA, USA) automated staining system along with Leica Reagents and the Refine DAB detection kit with Heat-Epitope-Retrieval-Buffer using the following antibodies and dilutions: AR, Biogenex USA, clone F39.4.1 (1:500); PSA, Dako, Denmark, clone ER-PR8 (1:4000); Racemase (AMACR), Dako, Germany, clone 13H4 (1:200); FASN, Abnova, UK, clone 3F2-1F3 (1:2000); PSMA, Dako, Germany, clone 3E6 (1:50); SCD1, Abcam, UK, polyclonal ab111301 (1:200); GSTP1, Goldenbridge Biotechnology, China, clone ZM-0110 (1:1000). Slides were counterstained with hematoxylin, dehydrated, and mounted. *ERG* translocation status was taken from a previous study [48].

### Gene expression data

Transcript profiling data were obtained by micro array experiments. Each malignant and non-malignant prostatectomy samples from 15 prostate cancer patients were analyzed (Table 1). Total RNA was isolated from frozen tissues according to the manufacturer's RNA extraction protocol (Qiagen, Hilden, Germany). The quantity and quality of isolated RNA were determined by a NanoDrop ND-1000 spectrophotometer (NanoDrop Technologies, Wilmington, DE, USA) and a Bioanalyzer 2100 (Agilent Technologies, Santa Clara, CA, USA). The RNA samples isolated from non-malignant as well as from malignant tumor tissue samples showed comparable median 260/280 absorbance ratios (2.01 and 2.00; Mann-Whitney-U-test; *P*=0.738) and median RIN values (7.75 and 8.25; Mann-Whitney–U-test; *P*=0.197). Samples with RNA integrity number (RIN) values >6 were used for mRNA expression analysis.

mRNA expression analysis was performed by one-color hybridizations on Human Genome U133 Plus 2.0 Arrays from Affymetrix (Santa Clara, CA, USA). After hybridization, microarrays were washed, scanned, and processed according to the supplier's protocol. The raw data were normalized using Genespring GX11 Software (Agilent Technologies, Santa Clara, CA, USA) with default parameters (MAS5 Summarization Algorithm, median of all samples as baseline transformation).

### Statistical analysis and data visualization

Prior to statistical analysis, log transformation of data was conducted to approach a normal distribution. The software tools R 2.8.0 (package nlme), R 3.0.2 (function coxph), TIBCO^®^ Spotfire^®^ 3.3.1, Genespring GX11 (Agilent), and GENESIS [49] were used for data analyses and visualisations.

Univariate statistical analyses were done by three mixed linear models (ANOVA) with subject as random intercept to account for individual baseline differences. First, for analysis of the metabolite differences between malignant and adjacent normal tissue samples, tissue type was included as a categorical fixed factor and BMI, age and storage time were included as numerical fixed factors. The interaction between tissue type and body mass index was taken into account to analyze BMI effects of tumor metabolism. Second, for analysis of the metabolite correlations with the Gleason score, a model with the fixed effects “Gleason score, body mass index, age and storage time” including the interaction between body mass index and Gleason score was applied. Third, for analysis of the metabolite correlations with the *ERG* translocation, a model with the fixed effects *ERG* translocation, body mass index, age and storage time including the interaction between body mass index and *ERG* translocation was applied. Correlation analysis of metabolite data versus protein expression levels was done within each tissue type using the Spearman method. Significance level was set to an alpha-error of 5%. The multiple testing problem was addressed by calculating the false-discovery rate (FDR) [50] with a q-value threshold of <0.2. Principal component analysis was done on the residuals from a mixed linear model with “subject” as random intercept in order to correct for inter-individual variability. The same residuals were used for hierarchical cluster analysis (average linkage), which provided a tree view of the distances between the metabolites expression profiles as well as the distance between tissue samples. Principal component analysis and cluster analysis were done on a subset of 76 subjects (≤10% missing values) in order to avoid effects due to imputation of missing values.

In Cox proportional hazards regression models standardized pool-normalized metabolite ratios of the tumor tissue as well as the tumor status (pT) and the categorized (<7, =7, >7) Gleason score were analyzed. The outcome of interest was the time until the clinical endpoint (biochemical (PSA) recurrence) was reached. Ties were dealt with by using Breslow's method. Correction for multiple comparisons was carried out as described earlier [50].

### Statement of significance

This large scale metabolomic profiling study of primary prostate cancers allowed for detailed sub-analyses in correlation with transcriptomic and proteomic data and is the first to demonstrate significant differences in the metabolome in dependence on the TMPRSS2-ERG translocation status.

## SUPPLEMENTARY MATERIAL FIGURES AND TABLES


