# Supplementary material for: Integration of tissue metabolomics, transcriptomics and immunohistochemistry reveals ERG- and gleason score-specific metabolomic alterations in prostate cancer
Source: Oncotarget. 2015 Nov 23;7(2):1421–38. doi: 10.18632/oncotarget.6370 (PMC4811470; doi:10.18632/oncotarget.6370)
Supplement: Supplementary file 1 [file oncotarget-07-1421-s001.pdf]

Integration of tissue metabolomics, transcriptomics and immunohistochemistry reveals *ERG*- and gleason score- specific metabolomic alterations in prostate cancer

Supplementary Material

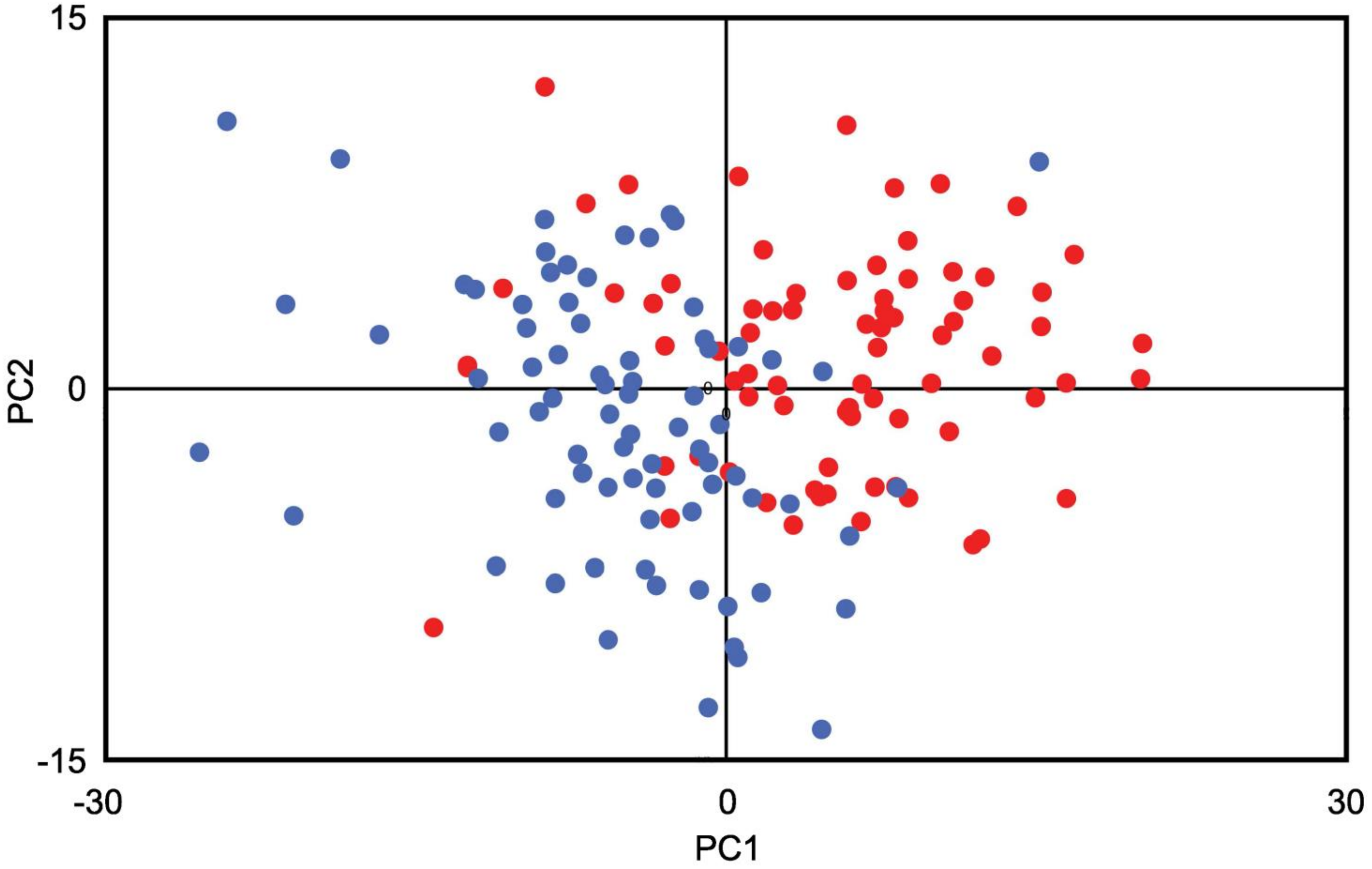

Supplemental Figure 1: Unsupervised analysis of 76 matched malignant and adjacent normal prostate tissue samples. Principal component analysis of subject-corrected residuals (fraction of variance captured by each principal component (PC): PC1: 30.3%; PC2: 9.8%) of MxP® data of malignant samples (red) and adjacent normal samples (blue).

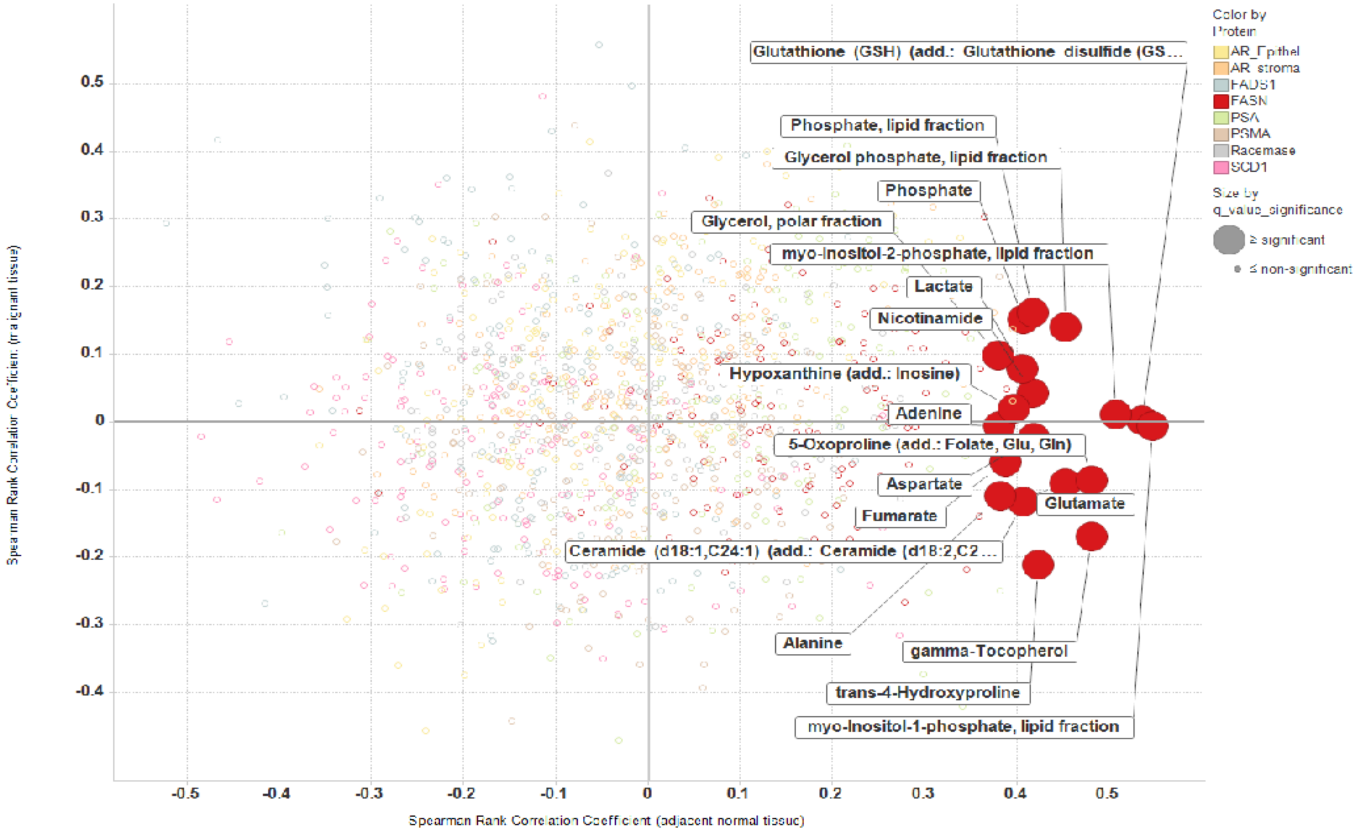

Supplemental Figure 2: Scatter plot of correlation coefficients of tissue-type specific Spearman rank correlation analysis of metabolite ratios versus protein expression data.

Supplemental Table 1: ANOVA results of carcinoma tissue versus adjacent normal as described in the method section. Ratio gives the fold change of carcinoma versus adjacent normal tissue.

Metabolite name	Ontology name	Ratio	p-value	FDR
2-Hydroxybehenic acid (C22:0)	Fatty acids, saturated	**4.38**	5.34E-15	4.52E-13
Cerebronic acid (2-OH-C24:0)	Fatty acids, hydroxylated	**3.49**	1.25E-12	3.52E-11
Tricosanoic acid (C23:0)	Fatty acids, saturated	**2.53**	1.04E-15	2.64E-13
Cystine	Amino acids, S-containing	**2.42**	6.50E-10	8.69E-09
Nicotineamide adenine dinucleotide (NAD)	Redox-carrier and related	**2.16**	4.80E-05	1.25E-04
Glycerophosphoethanolamine, polar fraction	Phospholipid metabolites	**2.07**	8.30E-11	1.26E-04
Uridine	Pyrimidine metabolism	**1.82**	5.40E-14	1.27E-04
alpha-Tocopherol	Tocopherols and related	**1.76**	3.12E-06	1.28E-04
Myristic acid (C14:0)	Fatty acids, saturated	**1.72**	6.86E-09	1.29E-04
Eicosenoic acid (C20:cis[11]1)	Fatty acids, mono-unsaturated	**1.66**	6.64E-07	1.30E-04
Hypoxanthine (additional: Inosine)	Purine metabolism	**1.65**	1.02E-13	1.31E-04
Eicosadienoic acid (C20:2) No 02	Fatty acids, poly-unsaturated	**1.64**	4.76E-09	1.32E-04
Dihydrocholesterol	Cholesterol and related	**1.61**	4.42E-05	1.33E-04
Xanthine	Purine metabolism	**1.59**	4.65E-07	1.34E-04
Spermidine	Polyamines	**1.59**	1.55E-06	1.35E-04
Biotin	C1-carriers and related	**1.58**	2.74E-15	1.36E-04
Glycerol-3-phosphate, polar fraction	Lipid precursors	**1.58**	1.71E-09	1.37E-04
Palmitoleic acid (C16:cis[9]1)	Fatty acids, mono-unsaturated	**1.58**	4.38E-07	1.38E-04
Isopentenyl pyrophosphate (IPP)	Mevalonate pathway	**1.57**	7.80E-11	1.39E-04
myo-Inositol-1-phosphate, lipid fraction (myo-Inositolphospholipids)	Phospholipid metabolites	**1.57**	2.02E-08	1.40E-04
myo-Inositol-2-phosphate, lipid fraction (myo-Inositolphospholipids)	Phospholipid metabolites	**1.56**	2.35E-06	1.41E-04
7-Methylguanine	Purine metabolism	**1.54**	2.20E-14	1.42E-04
Pentadecanol	Fatty alcohols	**1.54**	2.49E-08	1.43E-04
beta-Carotene	Carotenoids	**1.53**	3.09E-06	1.44E-04
Fructose	Glycolysis/Gluconeogenesis	**1.52**	2.98E-06	1.45E-04
Cysteine (additional: Cystine)	Amino acids, S-containing	**1.51**	4.21E-12	1.46E-04
2-Aminoadipic acid	Amino acid metabolites	**1.5**	8.35E-07	1.47E-04
Glycerol-2-phosphate	Miscellaneous	**1.49**	4.03E-10	1.48E-04
Heptadecanoic acid (C17:0)	Fatty acids, saturated	**1.48**	3.27E-09	1.49E-04

Eicosanoic acid (C20:0)	Fatty acids, saturated	**1.48**	6.24E-07	1.50E-04
erythro-Dihydrosphingosine (d16:0)	Sphingolipids	**1.47**	2.34E-06	1.51E-04
Aspartate	Amino acids, acidic	**1.45**	1.42E-10	1.52E-04
N-Acetylneuraminic acid	Aminosugars	**1.43**	1.95E-05	1.53E-04
4-Hydroxysphinganine (t18:0, Phytosphingosine), total	Sphingolipids	**1.43**	1.66E-04	1.54E-04
gamma-Tocopherol	Tocopherols and related	**1.42**	1.52E-07	1.55E-04
Glycerol, lipid fraction	Fatty alcohols	**1.39**	2.45E-08	1.56E-04
Cholestenol No 02	Cholesterol and related	**1.39**	3.46E-07	1.57E-04
Behenic acid (C22:0)	Fatty acids, saturated	**1.39**	5.76E-07	1.58E-04
Oleic acid (C18:cis[9]1)	Fatty acids, mono-unsaturated	**1.38**	2.28E-06	1.59E-04
Proline	Amino acids, neutral	**1.37**	3.06E-09	1.60E-04
Glucose, lipid fraction	Glycolipids	**1.37**	2.72E-04	1.61E-04
Ribose	Nucleobase related saccharides	**1.36**	7.26E-08	1.62E-04
Ornithine (additional: Arginine, Citrulline)	Urea cycle and related	**1.36**	2.14E-06	1.63E-04
Histidine	Amino acids, basic	**1.36**	4.60E-06	1.64E-04
14-Methylhexadecanoic acid	Fatty acids, branched	**1.36**	1.22E-05	1.65E-04
S-Adenosylmethionine	Methyl cycle	**1.35**	1.70E-04	1.66E-04
Elaidic acid (C18:trans[9]1)	Fatty acids, mono-unsaturated	**1.35**	5.56E-03	1.67E-04
Glycine	Amino acids, neutral	**1.34**	5.82E-12	1.68E-04
Docosapentaenoic acid (C22:cis[7,10,13,16,19]5)	Fatty acids, poly-unsaturated	**1.34**	2.37E-08	1.69E-04
Linoleic acid (C18:cis[9,12]2)	Fatty acids, poly-unsaturated	**1.33**	2.90E-08	1.70E-04
Palmitic acid (C16:0)	Fatty acids, saturated	**1.33**	1.77E-06	1.71E-04
Uracil	Pyrimidine metabolism	**1.32**	7.97E-14	1.72E-04
Hypoxanthine, lipid fraction	Miscellaneous	**1.32**	6.03E-06	1.73E-04
Xylitol	Polyols	**1.32**	6.12E-05	1.74E-04
Methionine	Amino acids, S-containing	**1.31**	5.49E-09	1.75E-04
Eicosapentaenoic acid (C20:cis[5,8,11,14,17]5)	Fatty acids, poly-unsaturated	**1.31**	5.98E-04	1.76E-04
trans-4-Hydroxyproline	Collagen metabolism	**1.3**	3.12E-10	1.77E-04
Fumarate	Citrate cycle	**1.3**	3.72E-07	1.78E-04
N-Acetylneuraminic acid, lipid fraction	Glycolipids	**1.3**	9.36E-05	1.79E-04
Isoleucine	Amino acids, branched chain	**1.29**	1.82E-10	1.80E-04
Pantothenic acid	Acyl-carriers and related	**1.29**	3.43E-06	1.81E-04
Malate	Citrate cycle	**1.29**	5.68E-06	1.82E-04
Sphingosine (d18:1) isomer No 03	Sphingolipids	**1.29**	1.15E-04	1.83E-04
Sphingomyelin (d18:1,C23:0)	Sphingomyelins	**1.28**	9.18E-14	1.84E-04
Docosahexaenoic acid (C22:cis[4,7,10,13,16,19]6)	Fatty acids, poly-unsaturated	**1.28**	6.51E-06	1.85E-04
Lignoceric acid (C24:0)	Fatty acids, saturated	**1.28**	8.68E-06	1.86E-04
3-O-Methylsphingosine (d18:1)	Sphingolipids	**1.28**	3.43E-05	1.87E-04

(additional: Sphingolipids, erythro-Sphingosine (d18:1), threo-Sphingosine (d18:1))				
erythro-Sphingosine-1-phosphate (d18:1)	Sphingolipids	**1.28**	2.41E-04	1.88E-04
Leucine	Amino acids, branched chain	**1.27**	2.58E-08	1.89E-04
Phenylalanine	Amino acids, aromatic	**1.27**	8.60E-08	1.90E-04
Threonine	Amino acids, neutral	**1.27**	2.91E-07	1.91E-04
Lysine	Amino acids, basic	**1.27**	8.75E-06	1.92E-04
Flavine adenine dinucleotide (FAD)	Redox-carrier and related	**1.27**	1.25E-04	1.93E-04
threo-Sphingosine (d18:1) (additional: Sphingolipids)	Sphingolipids	**1.27**	4.98E-04	1.94E-04
Glutamate	Amino acids, acidic	**1.26**	1.22E-08	1.95E-04
Tyrosine	Amino acids, aromatic	**1.26**	4.30E-06	1.96E-04
Glutamine	Amino acids, basic	**1.26**	1.19E-05	1.97E-04
5-O-Methylsphingosine (d18:1) (additional: Sphingolipids, erythro-Sphingosine (d18:1), threo-Sphingosine (d18:1))	Sphingolipids	**1.26**	1.51E-04	1.98E-04
Citrulline	Urea cycle and related	**1.25**	4.62E-06	1.99E-04
Glycerol phosphate, lipid fraction	Phospholipid metabolites	**1.25**	1.67E-05	2.00E-04
dihomo-gamma-Linolenic acid (C20:cis[8,11,14]3)	Fatty acids, poly-unsaturated	**1.25**	5.65E-05	2.01E-04
erythro-Sphingosine (d18:1) (additional: Sphingolipids)	Sphingolipids	**1.24**	6.61E-05	2.02E-04
Phosphate (inorganic and from organic phosphates)	Miscellaneous	**1.23**	5.05E-08	2.03E-04
Succinate	Citrate cycle	**1.23**	2.61E-07	2.04E-04
Stearic acid (C18:0)	Fatty acids, saturated	**1.23**	7.92E-06	2.05E-04
myo-Inositol, lipid fraction	Glycolipids	**1.22**	1.87E-03	2.06E-04
5-Oxoproline (additional: Folic acid, Glutamate, Glutamine)	Amino acid metabolites	**1.21**	5.52E-09	2.07E-04
Tryptophan	Amino acids, aromatic	**1.21**	3.43E-06	2.08E-04
Lysophosphatidylcholine (C18:2)	Lysophosphatidylcholines	**1.21**	1.47E-05	2.09E-04
Hypotaurine	Amino acid metabolites	**1.21**	2.97E-03	2.10E-04
Ribulose-5-phosphate	Pentose phosphate pathway	**1.21**	6.77E-03	2.11E-04
Homoserine	Amino acid metabolites	**1.2**	2.04E-06	2.12E-04
Nervonic acid (C24:cis[15]1)	Fatty acids, mono-unsaturated	**1.2**	8.15E-05	2.13E-04
Serine	Amino acids, neutral	**1.2**	1.15E-04	2.14E-04
Valine	Amino acids, branched chain	**1.19**	9.78E-06	2.15E-04
Guanosine	Purine metabolism	**1.19**	7.74E-05	2.16E-04
Arginine	Amino acids, basic	**1.19**	1.15E-04	2.17E-04
erythro-Dihydrosphingosine (d18:0)	Sphingolipids	**1.19**	4.02E-03	2.18E-04
Alanine	Amino acids, neutral	**1.18**	2.40E-05	2.19E-04

Adenine, lipid fraction	Miscellaneous	**1.18**	3.44E-02	2.20E-04
Threonic acid	Ascorbic acid and related	**1.17**	2.59E-03	2.21E-04
Pyrophosphate (PPi) (additional: Phosphate (inorganic and from organic phosphates))	Miscellaneous	**1.17**	8.61E-03	2.22E-04
Cytosine (additional: 2'-Deoxycytidine)	Pyrimidine metabolism	**1.16**	1.62E-08	2.23E-04
Pseudouridine	Pyrimidine metabolism	**1.16**	2.72E-04	2.24E-04
Phosphate, lipid fraction	Phospholipid metabolites	**1.15**	3.06E-04	2.25E-04
Sarcosine	Methyl cycle	**1.15**	2.60E-02	2.26E-04
Adenine	Purine metabolism	**1.14**	6.29E-06	2.27E-04
Glycerol, polar fraction	Lipid precursors	**1.13**	1.17E-04	2.28E-04
Nicotinamide	Redox-carrier and related	**1.13**	2.74E-04	2.29E-04
Cholesterol, total	Cholesterol and related	**1.13**	6.15E-04	2.30E-04
Glutathione (GSH) (additional: Glutathione disulfide (GSSG))	Redox-carrier and related	**1.13**	1.21E-03	2.31E-04
DAG (C18:1,C18:2)	Diacylglycerols	**1.13**	9.92E-03	2.32E-04
Pyruvate (additional: Phosphoenolpyruvate (PEP))	Glycolysis/Gluconeogenesis	**1.12**	4.81E-04	2.33E-04
Serine, lipid fraction	Miscellaneous	**1.12**	9.57E-03	2.34E-04
Coenzyme Q10	Redox-carrier and related	**1.1**	4.39E-02	2.35E-04
Lysophosphatidylcholine (C20:4)	Lysophosphatidylcholines	**1.08**	7.25E-03	2.36E-04
Arachidonic acid (C20:cis[5,8,11,14]4)	Fatty acids, poly-unsaturated	**1.07**	2.60E-02	2.37E-04
Dodecanol	Fatty alcohols	**1.07**	4.53E-02	2.38E-04
Ceramide (d18:1,C24:1) (additional: Ceramide (d18:2,C24:0))	Ceramides	**1.06**	8.55E-03	2.39E-04
2-Hydroxybutyrate	Energy metabolism, miscellaneous	**1.05**	2.70E-02	2.40E-04
Phosphatidylcholine No 02	Phosphatidylcholines	**0.96**	2.83E-02	2.41E-04
Creatine	Creatine metabolism	**0.93**	1.11E-02	2.42E-04
myo-Inositol	Polyols	**0.91**	4.07E-03	2.43E-04
Phosphatidylcholine (C18:0,C20:4)	Phosphatidylcholines	**0.89**	3.36E-05	2.44E-04
Phosphatidylcholine (C18:0,C22:6)	Phosphatidylcholines	**0.86**	2.34E-04	2.45E-04
Glutathione disulfide (GSSG)	Redox-carrier and related	**0.85**	9.29E-03	2.46E-04
Glucuronic acid	Sugar acids	**0.72**	1.07E-03	2.47E-04
cis-Aconitate (additional: Citrate)	Citrate cycle	**0.71**	7.58E-03	2.48E-04
Putrescine (additional: Agmatine)	Polyamines	**0.71**	9.77E-03	2.49E-04
Glucose	Monosaccharides	**0.7**	1.68E-03	2.50E-04
Gluconic acid (additional: Gluconolacton)	Sugar acids	**0.69**	6.87E-04	2.51E-04
Spermine	Polyamines	**0.69**	6.22E-03	2.52E-04
Maltose	Disaccharides	**0.65**	4.42E-06	2.53E-04

Maltotriose	Trisaccharides	**0.6**	1.03E-04	2.54E-04

Supplemental Table 2: Significantly changed protein expression data of 41 matched malignant and adjacent normal prostate tissue samples. Statistical analysis was done via a two-sided paired Wilcoxon signed rank test, the significance level was set to $p < 0.05$.

Analysis	Protein	Direction of change	p-value of paired Wilcoxon signed rank test
Malignant versus adjacent normal	AR (Epithelium)	increase	0.0260
	AR (Stroma)	decrease	0.0171
	PSA	decrease	< 0.0001
	Racemase	increase	< 0.0001
	FASN	increase	0.0001
	PSMA	increase	0.0009
	SCD1	increase	0.0292
	GSTP1	decrease	< 0.0001
